# A Short Essay on Insanity

**Published:** 1905-03

**Authors:** 


**A Short Essay on Insanity.** By CHARLES WILLIAMS. Pp. 22. London: H. J. Glaisher. 1905.

This elementary essay may be welcomed as one more effort to bring into prominence the importance of recognising the earliest symptoms of insanity. The author has treated the subject from a popular point of view, and in a simple, clear manner which can be readily understood by the unlearned in medical matters; and it is chiefly by the education of the public mind in this respect that the goal—viz. the warding off the approach of insanity—is to be attained, for it does not generally happen that a patient comes under medical care until the premonitory (and most curable) stage is passed, though even if such a "borderland" case does come under medical observation, the course is not so easy as the author implies. He says, "He or she should be at once placed under care and treatment." But alas! he or she may not be—and generally is not—willing, and however desirable such compulsory care and treatment may be, it has not as yet been proved sufficiently so to the authorities.

In a later stage, when actual delusion is present, we read (Part II.) of "the supreme importance of eradicating, or at least attempting to eradicate, a delusion at its first inception," and "the delusion at this stage may often be successfully removed." This sounds somewhat optimistic, and brings to mind many attempts to "eradicate delusion by reasonable argument" with as many failures, but possibly these failures prove conclusively the necessity for further efforts such as the essay advocates.

